# Plant-based diets and risk of frailty in community-dwelling older adults: the Seniors-ENRICA-1 cohort

**DOI:** 10.1007/s11357-022-00614-3

**Published:** 2022-07-04

**Authors:** Javier Maroto-Rodriguez, Mario Delgado-Velandia, Rosario Ortolá, Adrián Carballo-Casla, Esther García-Esquinas, Fernando Rodríguez-Artalejo, Mercedes Sotos-Prieto

**Affiliations:** 1 Department of Preventive Medicine and Public Health, School of Medicine, Universidad Autónoma de Madrid and IdiPaz (Instituto de Investigación Sanitaria Hospital Universitario La Paz), Calle del Arzobispo Morcillo, 4, 28029 Madrid, Spain; 2 CIBERESP (CIBER of Epidemiology and Public Health), Av. Monforte de Lemos, 3-5, 28029 Madrid, Spain; 3 IMDEA-Food Institute, CEI UAM+CSIC, Ctra. de Canto Blanco 8, E. 28049, Madrid, Spain; 4 Department of Environmental Health, Harvard T.H. Chan School of Public Health, 665 Huntington Avenue, Boston, MA 02115 USA

**Keywords:** Diet quality, Healthy aging, Physical impairment, Elderly, Pro-vegetarian pattern

## Abstract

Frailty is a geriatric syndrome that leads to increased risk of hospitalization, disability, and death. The effect of plant-based diets defined by the quality of their plant foods is unclear. Our objective is to study the association between two plant-based diet indices and the occurrence of frailty among community-dwelling older adults in Spain. We analyzed data from 1880 individuals aged ≥ 60 years from the Spanish Seniors ENRICA-1 cohort. We used a validated diet history to build two indices: (a) the healthful Plant-based Diet Index (hPDI) where healthy plant foods received positive scores, whereas less-healthy plant foods and animal foods received reverse scores; and (b) the unhealthful Plant-based Diet Index (uPDI), with positive scores to less-healthy plant foods and reverse scores to animal and healthy plant foods. Incident frailty was defined with the Fried phenotype. Study associations were summarized with odds ratios (OR) and 95% confidence intervals (CI) obtained from multivariable logistic models. After 3.3 years of follow-up, 136 incident frailty cases were ascertained. Comparing the highest vs. the lowest tertile of adherence, the OR [95% CI] for frailty was 0.43 (0.25–0.74; *p-*trend = .003) for the hPDI, and 2.89 (1.73–4.84; *p-*trend < .001) for the uPDI. Higher consumption of healthy plant foods was inversely associated with frailty (0.39 [0.23–0.66; *p*-trend < 0.001]); higher consumption of unhealthy plant foods was associated with higher frailty risk (2.40 [1.23–4.71; *p*-trend = .01]). In older adults, the hPDI was associated with lower risk of frailty, while the opposite was found for the uPDI.

**Supplementary Information:**

The online version contains supplementary material available at 10.1007/s11357-022-00614-3.

## Introduction

The world is aging rapidly. Older adults (≥ 60 years) were 1 billion people in 2019, and this number will increase by 34% in 2030 [1]. Consequently, health systems will face a growingly burden of age-related issues in the next years. One of the biggest threats to healthy aging is the frailty condition. Frailty is a geriatric syndrome that confers high vulnerability to stressors and leads to an increased risk of adverse health outcomes like disability, hospitalization, or death [2]. However, frailty is preventable and potentially reversible through interventions such as increasing physical activity or adequate nutrition [3–5].

There is growing interest on using diet to prevent frailty. So far, the available evidence suggests that higher consumption of fruits and vegetables [6–8] and higher adherence to diets rich in fruits, vegetables, legumes, and grains, and poor in red and processed meat such as the Mediterranean diet [9–12], the Baltic Sea Diet, and others [13–15] are all associated to lower risk of frailty.

The Plant-based Diet Indices (PDI), such as the healthful (hPDI) and the unhealthful (uPDI) indices, assess adherence to diets characterized by high consumption of plant-derived foods and lower consumption of animal-derived foods [16]. Although the hPDI share common characteristics with other healthy diets scores, unlike vegetarian diets, the hPDI focus on the quality of plant foods by assigning either positive scores to healthy plant foods (e.g., fruits, legumes, nuts) or reverse scores to less healthy plant foods (e.g., potatoes, sugar-sweetened beverages). In addition, animal-derived foods, while not excluded like in vegetarian diets, score inversely, even those typically associated to better health such as fish or dairy.

These novel indices have been associated with diabetes [16, 17], high blood pressure [18], cardiovascular disease [19], and mortality [20]. However, since their relationship with frailty has hardly ever been evaluated—and not in a Mediterranean population, we aimed to study the association between these two plant-based diet indices and the occurrence of frailty among community-dwelling older adults in Spain.

## Methods

### Study design and population

The Seniors ENRICA-1 is a cohort comprising 3289 non-institutionalized Spanish adults aged ≥ 60 years, recruited between 2008 and 2010 using multistage stratified random sampling and followed up in 2012. Details on the methodology have been previously reported [21]. Briefly, trained personnel performed sequentially: (1) a computer-assisted telephone interview about sociodemographic characteristics, health behaviors, and physician-diagnosed morbidity; (2) a home visit to collect biological samples that were sent to a central laboratory; (3) a second home visit to perform a physical examination and a computer-assisted face-to-face diet history.

The Seniors ENRICA-1 study was approved by the Clinical Research Ethics Committee of *La Paz* University Hospital in Madrid (study registration: NCT02804672). Participants provided written informed consent.

### Study variables

#### Plant-based diets

Food consumption was obtained using a validated computerized face-to-face diet history (DH-ENRICA) developed from that used in the European Prospective Investigation into Cancer and Nutrition (EPIC) cohort study in Spain (22). Standard food composition tables were used to estimate nutrient and energy intake [22].

We calculated the hPDI and the uPDI as described by Satija et al. [16] (Online Resource 1). We allocated each food consumed into 18 food groups under three blocks: healthy plant foods (whole grains, fruits, vegetables, nuts, legumes, vegetable oils, and coffee and tea); unhealthy plant foods (fruit juices, sugar-sweetened beverages, refined grains, potatoes, and sweets and desserts), and animal foods (animal fats, dairy, eggs, fish and seafood, meat, and miscellaneous animal-based foods). Each food group was divided into quintiles of consumption and scored between one (worst) and five (best). For the hPDI, healthy plant foods were given positive scores, while the unhealthy plant and animal foods were given reverse scores. For the uPDI, positive scores were given to unhealthy plant foods, and reverse scores to healthy plant and animal foods (Online Resource 2). The food groups were summed to obtain the indices. The scores range was 18 to 90.

#### Frailty assessment

Incident frailty was defined, according to Fried et al. [23], as developing at least three out of the following five criteria at follow-up (2012): (1) Exhaustion, responding affirmatively to any of these two statements adapted from the Center for Epidemiologic Studies Depression Scale [24]: “I feel that anything I do is a big effort” or “I feel that I cannot keep on doing things”, ≥ 3–4 days/week; (2) Low physical activity, walking ≤ 2.5 h/week (men) or 2 h/week (women) [25]; (3) Slow gait speed, being in the lowest cohort-specific quintile in a 2.44-m walking speed test adjusted for sex and height [26]; (4) Unintentional weight loss, defined as unintentional loss of ≥ 4.5 kg in the preceding year; and (5) Muscle weakness, defined as being in the lowest cohort-specific quintile of grip strength, adjusted for sex and BMI; strength was assessed as the highest of two consecutive measures in the dominant hand with a Jamar dynamometer [27].

#### Potential confounders

At baseline, participants reported their sex, age, educational level (primary or less, secondary, university), and other possible confounding factors: smoking status (never, former, current), recreational physical activity (Metabolic Equivalents of task-hours/week, METs*h/wk), energy intake (kcal/day), physician-diagnosed diseases (type 2 diabetes mellitus, cardiovascular disease, chronic lung disease, osteomuscular disease, cancer, and depression), number of medications taken (0, 1–3, 4–6, > 6; including any of the following: low-dose aspirin, beta-blockers, blockers of the renin-angiotensin system, diuretics, statins, insulin, oral antidiabetic drugs, antiplatelet medication, anticoagulant medication, and sleeping pills), and alcohol consumption (servings/day).

Hours of leisure-time physical activity were obtained with the Spanish-validated version of the EPIC-cohort questionnaires [28]. Energy and alcohol intake were estimated from the diet history. Alcohol serving was expressed as any volume of a drink containing 10 g of pure alcohol. Finally, the body mass index (BMI) was calculated as weight (kg) divided by squared height (m), both measured during the physical examination.

### Statistical analyses

From the 3289 initial participants, we excluded at baseline 154 participants with any missing frailty data, 25 with missing BMI, 7 with implausible energy intakes (men: < 800 kcal or > 5000; women: < 500 kcal or > 4000 kcal), and 41 participants who were already frail. At the follow-up, in 2012, we excluded those who died (108), those who were lost to follow-up (662), and 412 with missing frailty data at this point. Therefore, the analytical sample comprised 1880 individuals (Online Resource 3). Participants’ characteristics were summarized using proportions for categorical variables and mean and standard deviation (SD) for continuous variables.

We used multivariable logistic regression models to evaluate associations between baseline tertiles of PDIs and incident frailty, which were summarized using odds ratios (OR) with 95% confidence intervals (CI). The lowest tertile of each PDI was used as reference. In addition, we evaluated associations between tertiles of the PDIs and the occurrence of each frailty criterion, in order to discern how these criteria behaved separately. Moreover, we assessed associations for healthy, unhealthy plant foods, and animal foods. Three progressively adjusted models were fit: Model 1, adjusted for sex, age, education, and alcohol intake; model 2, further adjusted for smoking status, BMI, energy intake, and physical activity; and model 3, further adjusted for chronic diseases and number of medications taken. We performed stratified analyses by sex and tested interaction terms to assess possible differential associations. No significant interaction was found, so we reported results for the total sample.

In secondary analyses: (1) We excluded pre-frail individuals (those meeting one or two frailty criteria) and participants with chronic diseases at baseline, as these conditions can lead to diet changes; (2) due to the close relation between frailty and poor health, we restricted the analyses to participants that self-reported good health and independence in instrumental activities of daily living (IADL); (3) we excluded “unintentional weight loss” from frailty criteria (the definition of frailty of ≥ 3 criteria remained unchanged), since a healthy diet could lead to weight changes [29]; (5) we built an alternative hPDI in which fish, eggs, and dairy products scored positively instead of reversely because these foods have been shown to be associated with healthy aging and lower frailty risk [30–32].

Statistical significance was established at two-sided *p* < 0.05. Analyses were performed using Stata, version 16.0 (Stata-Corp LLC, College Station, TX).

## Results

Participants had a mean (SD) age of 68.7 (6.4) years, and 51.7% were women; corresponding figures were 59.7 (5.6) for the hPDI and 54.9 (5.3) for the uPDI. Compared to participants in the lowest tertile of the hPDI, those in the highest tertile were more likely to be women, non-smokers, to have lower BMI and less energy and alcohol intake, and to be more physically active, whereas those in the highest tertile of the uPDI were more often women and non-smokers, had lower education and energy intake (Table 1). Compared to participants in the analytical sample, those lost to follow-up or excluded due to missing frailty data were more likely to be women, have lower education, and have a BMI ≥ 30 (Online Resource 4).

**Table 1:** Baseline characteristics of older adults participating in the Seniors ENRICA-1 cohort by tertiles of plant-based diet indices (*N* = 1880)

	Healthful Plant-based Diet Index			Unhealthful Plant-based Diet Index			Total	
	Tertile 1 (lowest) 41–55 p	Tertile 2 56–61 p	Tertile 3 (highest) 62–80 p	Tertile 1 (lowest) 37–54 p	Tertile 2 55–59 p	Tertile 3 (highest) 60–75 p		
*n* (%)	429 (22.82)	765 (40.69)	686 (36.49)	879 (46.76)	639 (33.99)	362 (19.26)	1880 (100)	
Index score, range (18–90), mean (SD)	52.43 (2.62)	58.60 (1.71)	65.56 (3.22)	50.32 (3.13)	56.83 (1.37)	62.38 (2.52)	hPDI 59.73 (5.63)	uPDI 54.85 (5.32)
Sex, women, *n* (%)	177 (41.26)	385 (50.33)	409 (59.62)	425 (48.35)	340 (53.21)	206 (56.91)	971 (51.65)	
Age, years, mean (SD)	67.86 (6.29)	69.33 (6.76)	68.38 (5.91)	68.02 (6.01)	68.79 (6.59)	69.93 (6.68)	68.65 (6.38)	
Education, *n* (%)								
≤ Primary	216 (50.35)	414 (54.12)	367 (53.50)	443 (50.40)	347 (54.30)	207 (57.18)	997 (53.03)	
Secondary	120 (27.97)	186 (24.31)	163 (23.76)	220 (25.03)	163 (25.51)	86 (23.76)	469 (24.95)	
University	93 (21.68)	165 (21.57)	156 (22.74)	216 (24.57)	129 (20.19)	69 (19.06)	414 (22.02)	
Smoking status, *n* (%)								
Current	62 (14.45)	97 (12.68)	62 (9.04)	102 (11.60)	72 (11.27)	47 (12.98)	221 (11.76)	
Former	137 (31.93)	233 (30.46)	207 (30.17)	299 (34.02)	195 (30.52)	83 (22.93)	577 (30.69)	
Never	230 (53.61)	435 (56.86)	417 (60.79)	478 (54.38)	372 (58.22)	232 (64.09)	1082 (57.55)	
BMI, kg/m^2^, *n* (%)								
< 25	71 (16.55)	150 (19.61)	147 (21.43)	174 (19.80)	118 (18.47)	76 (20.99)	368 (19.57)	
25–29.9	212 (49.42)	391 (51.11)	334 (48.69)	430 (48.92)	338 (52.90)	169 (46.69)	937 (49.69)	
≥ 30	146 (34.03)	224 (29.28)	205 (29.88)	275 (31.29)	183 (28.64)	117 (32.32)	575 (30.59)	
Energy intake, kcal/day, mean (SD)	2335 (550)	2053 (554)	1815 (499)	2142 (566)	1971 (545)	1863 (559)	2031 (569)	
Alcohol intake, servings/day, mean (SD)	1.35 (2.09)	1.03 (1.74)	0.89 (1.61)	1.06 (1.70)	1.07 (1.82)	0.99 (1.94)	1.05 (1.79)	
Physical activity, METs*h/wk, mean (SD)	20.97 (15.82)	21.78 (15.32)	22.29 (15.16)	22.78 (15.45)	20.77 (14.20)	21.14 (17.01)	21.78 (15.38)	
Prevalent diseases, *n* (%)								
Cardiovascular disease^a^	22 (5.13)	39 (5.10)	36 (5.25)	45 (5.12)	34 (5.32)	18 (4.97)	97 (5.16)	
Type 2 diabetes mellitus	74 (17.25)	117 (15.29)	93 (13.56)	129 (14.68)	103 (16.12)	52 (14.36)	284 (15.11)	
Cancer	9 (2.10)	13 (1.70)	12 (1.75)	18 (2.05)	8 (1.25)	8 (2.21)	34 (1.81)	
Chronic lung disease^b^	24 (5.59)	68 (8.89)	51 (7.43)	63 (7.17)	47 (7.36)	33 (9.12)	143 (7.61)	
Osteomuscular disease^c^	188 (43.82)	366 (47.84)	339 (49.42)	409 (46.53)	311 (48.67)	173 (47.79)	893 (47.50)	
Depression	33 (7.69)	51 (6.67)	54 (7.87)	60 (6.83)	44 (6.89)	34 (9.39)	138 (7.34)	
Medicines per day, *n* (%)								
0	121 (28.21)	202 (26.41)	181 (26.38)	236 (26.85)	167 (26.13)	101 (27.90)	504 (26.81)	
1–3	233 (54.31)	399 (52.16)	361 (52.62)	469 (53.36)	342 (53.52)	182 (50.28)	993 (52.82)	
4–6	68 (15.85)	146 (19.08)	118 (17.20)	142 (16.15)	120 (18.78)	70 (19.34)	332 (17.66)	
> 6	7 (1.63)	18 (2.35)	26 (3.79)	32 (3.64)	10 (1.56)	9 (2.49)	51 (2.71)	

Abbreviations: *BMI* body mass index, *p* points, *SD* standard deviation

^a^Including myocardial infarction, stroke, and heart failure

^b^Including asthma and chronic obstructive pulmonary disease

^c^Including osteoarthritis, rheumatoid arthritis, and hip fracture

### PDIs and frailty risk

We ascertained 136 frailty cases after a mean follow-up of 3.3 years. Study participants showed an inverse dose–response relationship between the hPDI and frailty: the full adjusted OR (95% CI) compared to the lowest of hPDI was 0.51 (0.31–0.84) for the second and 0.43 (0.25–0.74) for the highest tertile (*p*-trend = 0.003). Conversely, the uPDI showed a direct dose–response with frailty, with OR (95% CI) of 1.81 (1.12–2.94) and 2.89 (1.73–4.84) respectively (*p*-trend < 0.001) (Table 2).

**Table 2:** Odds ratios (95% CI) for the occurrence of frailty by tertiles of plant-based diet indices and tertiles of total servings of food groups consumed, over 3.3 years of follow-up in the Seniors ENRICA-1 cohort (*N* = 1880)

Plant-based diet indices or food groups consumed				
	Tertile 1 (lowest)	Tertile 2	Tertile 3 (highest)	*p*-trend
Healthful Plant-based Diet Index				
	41–55 p	56–61 p	62–80 p	
* n*/*N*	30/309	54/701	52/870	
Model 1^a^	Ref	0.51 (0.32–0.81)	0.44 (0.27–0.71)	**0.001**
Model 2^b^	Ref	0.51 (0.32–0.84)	0.42 (0.25–0.71)	**0.002**
Model 3^c^	Ref	0.51 (0.31–0.84)	0.43 (0.25–0.74)	**0.003**
Unhealthful Plant-based Diet Index				
	37–54 p	55–59 p	60–75 p	
* n*/*N*	34/764	55/772	47/344	
Model 1^a^	Ref	1.71 (1.08–2.68)	2.68 (1.68–4.29)	** < 0.001**
Model 2^b^	Ref	1.80 (1.12–2.89)	2.84 (1.72–4.71)	** < 0.001**
Model 3^c^	Ref	1.81 (1.12–2.94)	2.89 (1.73–4.84)	** < 0.001**
Healthy plant foods^d^				
* n*/*N*	45/349	54/701	37/830	
Model 1^a^	Ref	0.65 (0.41–1.01)	0.43 (0.27–0.69)	**0.001**
Model 2^b^	Ref	0.70 (0.43–1.12)	0.40 (0.23–0.67)	**0.001**
Model 3^c^	Ref	0.71 (0.44–1.15)	0.39 (0.23–0.66)	** < 0.001**
Unhealthy plant foods^e^				
* n*/*N*	49/717	52/737	35/426	
Model 1^a^	Ref	1.18 (0.77–1.81)	1.74 (1.07–2.83)	**0.032**
Model 2^b^	Ref	1.50 (0.93–2.42)	2.40 (1.24–4.64)	**0.009**
Model 3^c^	Ref	1.52 (0.93–2.48)	2.40 (1.23–4.71)	**0.010**
Animal foods^f^				
* n*/*N*	56/704	47/677	33/499	
Model 1^a^	Ref	0.98 (0.64–1.50)	1.20 (0.75–1.94)	0.502
Model 2^b^	Ref	0.85 (0.54–1.35)	0.95 (0.54–1.66)	0.786
Model 3^c^	Ref	0.81 (0.51–1.30)	0.89 (0.50–1.57)	0.618

Abbreviations: *BMI* body mass index, *CI* confidence interval, *p* points, *Ref.* reference

^a^Model 1: Adjusted for sex, age, educational level (primary, secondary, university), alcohol consumption

^b^Model 2: Model 1 + smoking status (current, former, never), BMI (< 25, 25–29.9, ≥ 30), energy intake, physical activity

^c^Model 3: Model 2 + and prevalent diseases (type 2 diabetes mellitus, cardiovascular disease [myocardial infarction, stroke, or heart failure], chronic lung disease [asthma or chronic obstructive pulmonary disease], osteomuscular disease [osteoarthritis, rheumatoid arthritis or hip fracture], cancer and depression), medicines consumption (0, 1–3, 4–6, > 6)

^d^Healthy plant foods: whole grains, fruits, vegetables, nuts, legumes, vegetable oils, and coffee/tea

^e^Unhealthy plant foods: fruit juices, sugar-sweetened beverages, refined grains, potatoes, and sweets/desserts

^f^Animal foods: fats, dairy products, eggs, fish and seafood, meat, and miscellaneous animal-based foods

Significance of bold entries is *p *< 0.05

The results remained robust in sensitivity analyses (Online Resource 5): (1) Excluding pre-frail participants (adjusted OR [95% CI] for the highest vs. lowest tertile: 0.40 [0.20–0.82] for hPDI, *p*-trend = 0.01; and 3.84 [1.96–7.50] for uPDI, *p*-trend < 0.001); (2) excluding participants with diagnosed chronic diseases (0.40 [0.18–0.88] for hPDI, *p*-trend = 0.02; and 3.03 [1.50–6.14] for uPDI, *p*-trend = 0.002); (3) among participants self-reportedly healthy and independent in IADL (0.29 [0.10–0.87] for hPDI, *p*-trend = 0.03; and 3.54 [1.28–9.78] for uPDI, *p*-trend = 0.01); (4) excluding “unintentional weight loss” from frailty criteria (0.53 [0.28–0.99], *p*-trend = 0.06 for hPDI; and 2.09 [1.17–3.74], *p*-trend = 0.01 for uPDI); (5) when using the alternative hPDI (0.40 [0.23–0.68], *p*-trend < 0.001).

### Food groups and frailty risk

**Fig. 1:**
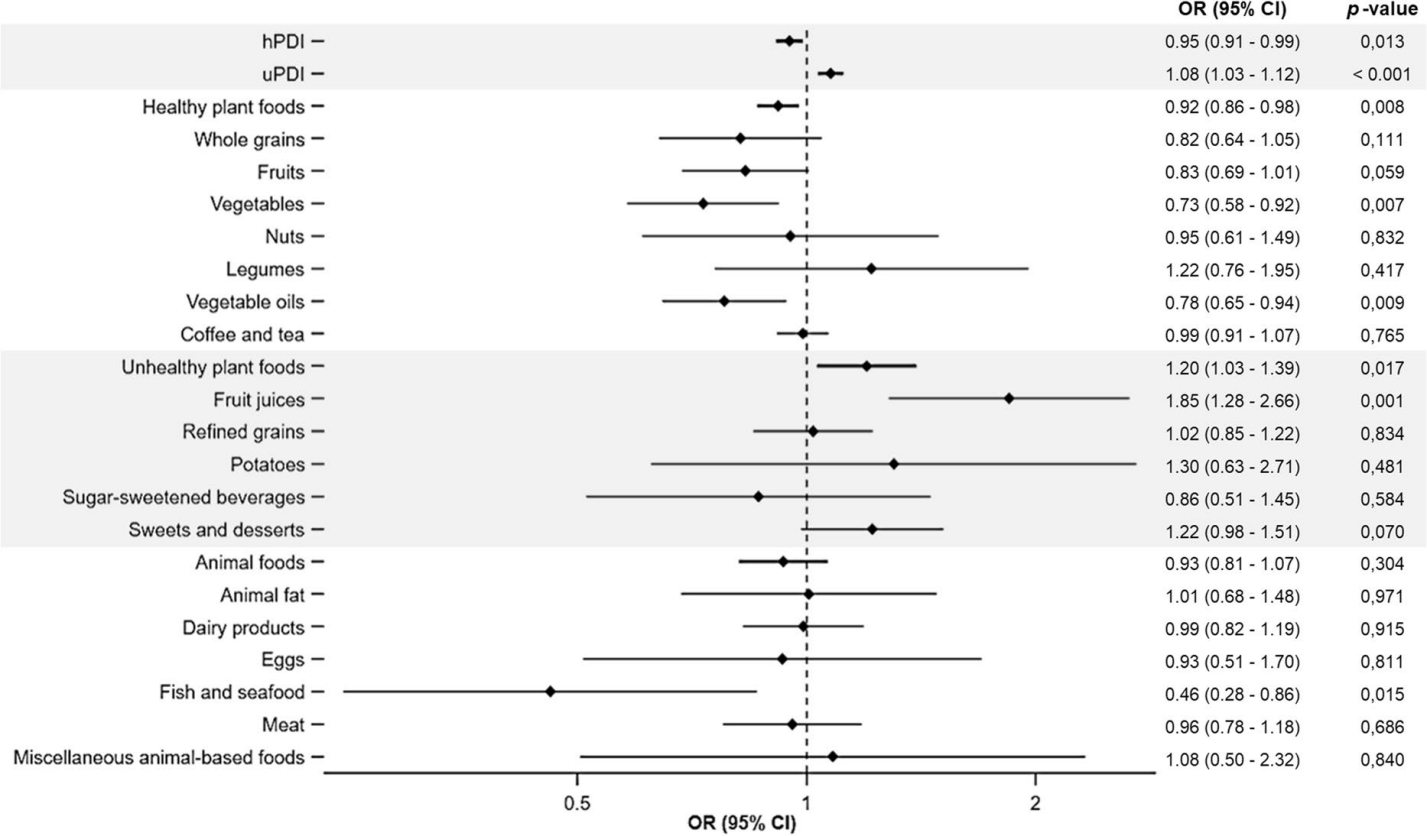
Odds ratio (95% CI) for the occurrence of frailty per each Plant-based Diet Index (1-unit increase), each food group, healthy plant food, unhealthy plant food, and animal food, over 3.3 years of follow-up in the Seniors ENRICA-1 cohort participants (*N* = 1880). Abbreviations: OR odds ratio, CI confidence interval, hPDI healthful Plant-based Diet Index, uPDI unhealthful Plant-based Diet Index. Adjusted for sex, age, educational level (primary, secondary, university), alcohol consumption, smoking status (current, former, never), BMI (< 25, 25–29.9, ≥ 30), energy intake, physical activity, prevalent diseases (type 2 diabetes mellitus, cardiovascular disease [myocardial infarction, stroke, or heart failure], chronic lung disease [asthma or chronic obstructive pulmonary disease], osteomuscular disease [osteoarthritis, rheumatoid arthritis or hip fracture], cancer and depression), number of medicines consumed (0, 1–3, 4–6, > 6)

Four out of the 18 food groups were independently associated with lower frailty risk: vegetables (OR per 1-serving increase: 0.73 [0.58–0.92]), vegetable oils (0.78 [0.65–0.94]), and fish and seafood (0.46 [0.28–0.86]), while fruit juices were associated with greater frailty risk (1.85 [1.28–2.66]) (Fig. 1). Most of the association between the other food groups with frailty were in the expected direction (note the associations of whole grains, fruits, or sweets and desserts).

In addition, we found an association between higher consumption of overall healthy plant food and lower risk of frailty (adjusted OR [95% CI] for highest vs. lowest tertile: 0.39 [0.23–0.66]; *p*-trend < 0.001). Conversely, higher consumption of unhealthy plant foods was associated with higher risk of frailty (2.40 [1.23–4.71]; *p*-trend = 0.01) (Table 2).

### PDIs and frailty criteria

When we analyzed the association between the Fried criteria and PDIs (Table 3), we found an association between the uPDI and three criteria: exhaustion (adjusted OR [95% CI] for highest vs. lowest tertile: 1.72 [1.19–2.50], *p*-trend = 0.005), slow walking speed (1.60 [1.13–2.28], *p*-trend = 0.003), and unintentional weight loss (1.89 [1.21–2.97], *p*-trend = 0.006).

**Table 3:** Odds ratios (95% CI) for the occurrence of each frailty criterion by tertiles of plant-based diet indices, over 3.3 years of follow-up in the Seniors ENRICA-1 cohort

Plant-based diet indices				
	Tertile 1 (lowest)	Tertile 2	Tertile 3 (highest)	*p*-trend
Exhaustion (*N* = 1880)				
*n*/*N*	53/429	109/765	89/686	
hPDI	Ref	0.99 (0.67–1.46)	0.83 (0.55–1.25)	0.307
*n*/*N*	92/879	86/639	73/362	
uPDI	Ref	1.19 (0.85–1.66)	1.72 (1.19–2.50)	**0.005**
Low physical activity (*N* = 1880)				
*n*/*N*	80/429	122/765	94/686	
hPDI	Ref	0.89 (0.63–1.24)	0.79 (0.55–1.14)	0.212
*n*/*N*	123/879	114/639	59/362	
uPDI	Ref	1.29 (0.96–1.73)	1.02 (0.70–1.47)	0.630
Low walking speed (*N* = 1855)				
*n*/*N*	75/423	107/755	97/677	
hPDI	Ref	0.75 (0.53–1.05)	0.85 (0.60–1.22)	0.486
*n*/*N*	103/872	109/626	67/357	
uPDI	Ref	1.54 (1.14–2.09)	1.60 (1.13–2.28)	**0.003**
Unintentional weight loss (*N* = 1858)				
*n*/*N*	41/425	60/755	47/678	
hPDI	Ref	0.79 (0.51–1.23)	0.70 (0.43–1.13)	0.150
*n*/*N*	55/865	52/633	41/360	
uPDI	Ref	1.34 (0.89–2.01)	1.89 (1.21–2.97)	**0.006**
Muscle weakness (*N* = 1877)				
*n*/*N*	132/428	275/764	244/ 685	
hPDI	Ref	1.03 (0.77–1.38)	1.06 (0.78–1.44)	0.700
*n*/*N*	279/878	230/637	142/362	
uPDI	Ref	1.01 (0.82–1.33)	1.01 (0.75–1.36)	0.883

Abbreviations: *BMI* body mass index, *CI* confidence interval, *hPDI* healthful Plant-based Diet Index, *Ref.* reference, *uPDI* unhealthful Plant-based Diet Index

All models: adjusted for sex, age, educational level (primary, secondary, university), alcohol consumption, smoking status (current, former, never), BMI (< 25, 25–29.9, ≥ 30), energy intake, physical activity, prevalent diseases (type 2 diabetes mellitus, cardiovascular disease [myocardial infarction, stroke, or heart failure], chronic lung disease [asthma or chronic obstructive pulmonary disease], osteomuscular disease [osteoarthritis, rheumatoid arthritis, or hip fracture], cancer, and depression), medicines consumption (0, 1–3, 4–6, > 6)

Significance of bold entries is *p* < 0.05

## Discussion

In this cohort of community-dwelling older adults in Spain, higher adherence to the hPDI was associated with lower frailty risk. On the contrary, higher adherence to the uPDI was associated with higher frailty risk. Results were in the same direction for robust, morbidity-free, IADL-independent participants, when “unintentional weight loss” was excluded from frailty criteria, and when some foods from animal origin scored positively in the hPDI (egg, fish, and dairy).

Our findings are of major importance because: (1) they highlight the differential health effect of consuming healthy plant-derived foods (e.g., vegetables, fruits, whole grains, nuts) over unhealthy plant-derived (e.g., refined grains, sugary beverages) and animal foods; (2) a healthful plant-based diet is aligned with the EAT-LANCET commission recommendations for the sustainability of food production systems, since this kind of diets are concordant with feasible dietary guidelines in most countries [33]; and (3) they shed light into the controversial perils of insufficient animal-based protein intake in older adults [34], as the hPDI does not exclude, but rather scores reversely animal food consumption.

To our knowledge, this is the first study that assesses the associations between plant-based diets and frailty in a Mediterranean older population. Nonetheless, our results are consistent with previous literature. One study in a cohort of 14,159 Chinese adults, aged 45–74 years, found that higher adherence to a healthy plant-based diet was associated with higher odds of healthy aging after 20 years (although the “healthy aging” criteria were different than the frailty phenotype used here) [35], and an analysis of pooled data from three European cohorts (Seniors ENRICA-1, 3C Bordeaux, and AMI) found an inverse dose–response relationship between consumption of fruits and vegetables and frailty risk [6]. Similar results were found in a cohort of US women aged > 60 years [8]. In addition to the protective effect of vegetables consumption, we also found an inverse association between vegetable oils and frailty. In Spain, the primary source of oil consumption is olive oil, in our cohort represents 90% of this group. In fact, a recent publication in the Seniors-ENRICA-1 cohort showed that the consumption of olive oil, especially virgin oil, was associated with lower risk of frailty (OR for ≈3 vs ≈1 tablespoons/day: 0.47 [0.29, 0.78]) in comparison with lower consumption [36].

In addition, in the same cohort, recent research showed that higher adherence to a Mediterranean lifestyle was associated with lower risk of frailty [37]. Specifically, the block of Mediterranean food consumption was independently associated with lower risk of frailty. Although the Mediterranean diet shares common components of the plant-based diet, they differ in some features such as the culinary traditions of the Mediterranean diet with the use of abundant extra-virgin olive oil and moderate wine drinking at meals (which is not included in the hPDI). Although, most of the functional properties of the plant-based foods included in both dietary patterns could explain the association with frailty, our results highlight that the quality of those plant-based foods is also important since unhealthy plant foods had the largest effect on frailty maybe due to the ultra-processed nature of most of the foods.

Our results for the uPDI and unhealthy plant foods are in the same direction of other studies suggesting that higher consumption of potatoes [38], sugar-sweetened beverages [39], and fruit juices [40] was associated with higher frailty risk.

Of note is that we did not find a clear association between animal-derived foods and frailty. This may be due to the myriad of foods included within this block, many of which have opposite effects. For instance, in the Seniors ENRICA-1 higher consumption of low-fat dairy products was inversely associated with frailty risk [41], and elevated intake of fish and blue fish was inversely associated with the accumulation of health deficits in older adults [30]; however, higher consumption of processed meat was associated with a higher frailty risk [10]. In fact, we found that a modified hPDI with positive scores to milk, eggs, and fish was more strongly and still inversely associated with frailty risk.

Finally, regarding each criterion of the frailty index, although the strength with which both indices were associated with frailty was similar, we found an inverse association only between the uPDI and some frailty criteria. This could indicate that the hPDI dietary pattern is most likely associated with the whole frailty syndrome since it better captures a patient’s overall health condition than its independent components.

Future studies should assess additional aspects for PDIs, such as its implementation along with caloric restrictions, different macronutrient balance, or changing time of eating; prior works have found that age-related impairments could be prevented with these strategies [42], and taking together, diet and its associated habits could complement each other to manage healthy aging.

### Plausible biological mechanisms

The protective effect of hPDI on frailty might be due to the nutrient intake from some of its components. For example, the antioxidant effects of vitamins C and E, carotenoids, and selenium from fruits and vegetables may protect against sarcopenia by reducing the exposure of muscle fibers to oxidative stress [43, 44]. In addition, the contribution of proteins from legumes and nuts could help prevent sarcopenia [44]. Other mechanisms underlying our findings could involve the potential anti-inflammatory effect of fruits, olive oil, unsaturated fatty acids, nuts, or coffee, which may help reduce the low-grade chronic inflammation associated with frailty [45–48].

Regarding the uPDI, the often high refined and added sugar content, but low in fiber, of its components might contribute to its detrimental association with frailty [49]. Low dietary fiber has been found to be correlated to higher postprandial glycemia [50]. If plasma glucose levels are maintained over time, they could be a risk factor for frailty in non-diabetic older adults [51]. In addition, these sugars are risk factors for health issues which have been also associated to higher frailty risk, like insulin resistance and metabolic syndrome [52] or dysregulation of the glucose hormones dynamics [53].

On the other hand, the food groups included in the uPDI are deficient in certain ingredients, being perhaps related to the subsequent frailty risk, too. Refined grains lack both germ and bran, which are the primary source of several phytochemicals and other antioxidants (e.g., flavonoids, zeaxanthin, lutein, and β-cryptoxanthin) [54]. Similarly, fruit juices lack phytochemicals and fiber that whole fruits hold. A diet rich in these foods rather than its complete counterparts might lack enough antioxidant and anti-inflammatory molecules, which would redound in long-term poorer physical health. However, it is certain that refined grains alone were not associated to frailty in our study, maybe because the individual effect of food groups is small when we account holistically for dietary patterns, and they were one of the primary sources of vegetal protein in a previous study using this cohort evaluating the role of vegetable vs. animal proteins and healthy aging [53]. Additionally, functional foods could play antagonic or summatory effects on health, for example modulating the epigenome [55] and ultimately health. Anyway, further research is needed to understand the underlying mechanisms of the PDIs-frailty association.

### Strengths and limitations

Strengths of this study include its prospective design, the use of measured data whenever possible (e.g., gait speed, grip strength, and BMI), and the reliance in validated tools when collecting self-reported information (e.g., food consumption was recorded with a validated diet history) [22]. Nevertheless, some limitations should be noted. First, self-reported data on diet, physical activity, and chronic diseases could lead to some misclassification and bias. However, if it were the case, it would most likely be non-differential, thus driving the study associations towards the null. Second, lifestyle was only assessed at one point; hence, we cannot evaluate the effect of lifestyle changes. Third, it is possible for a healthy diet to be framed in a healthy lifestyle that also influences frailty risk. Fourth, the analytical sample came from community-dwelling older adults; therefore, our results are not generalizable to institutionalized older adults. Finally, the presence of residual confounding cannot be ruled out; however, we did adjust for the most relevant confounders. Thus, further adjustment would not likely change the direction of associations.

## Conclusion

In this cohort of community-dwelling Spanish older adults, higher adherence to the hPDI was associated with lower frailty risk, while the opposite was found for the uPDI. These results should be confirmed on other older populations within and without the Mediterranean basin; if this is the case, older adults adhering to a diet rich in healthy plants could be reassured that their diet protects them from physical impairment and frailty.

## Supplementary Information

Below is the link to the electronic supplementary material.

Supplementary file1 (DOCX 52 KB)
