# Supplementary material for: Plant-based diets and risk of frailty in community-dwelling older adults: the Seniors-ENRICA-1 cohort
Source: GeroScience. 2022 Jul 4;45(1):221–32. doi: 10.1007/s11357-022-00614-3 (PMC9886709; doi:10.1007/s11357-022-00614-3)
Supplement: Supplementary file 1 — Supplementary file1 (DOCX 52 KB) [file 11357_2022_614_MOESM1_ESM.docx]

**Title**: Plant-based diets and risk of frailty in community-dwelling older adults: the Seniors-ENRICA-1 cohort.

**Authors**: Javier Maroto-Rodriguez, MSc^1^; Mario Delgado-Velandia MSc^1,2^; Rosario Ortolá, PhD^1,2^; Adrián Carballo-Casla MSc^1,2^; Esther García-Esquinas, PhD^1,2^; Fernando Rodríguez-Artalejo, MD^1,2,3^; Mercedes Sotos-Prieto PhD^1,2,3,4^

^1^ Department of Preventive Medicine and Public Health. School of Medicine. Universidad Autónoma de Madrid and IdiPaz (Instituto de Investigación Sanitaria Hospital Universitario *La Paz*), Calle del Arzobispo Morcillo, 4. 28029, Madrid, Spain.

^2^ CIBERESP (CIBER of Epidemiology and Public Health), Av. Monforte de Lemos, 3-5. 28029, Madrid, Spain.

^3^ IMDEA-Food Institute. CEI UAM+CSIC, Ctra. de Canto Blanco 8, E. 28049, Madrid, Spain.

^4^ Department of Environmental Health, Harvard T.H. Chan School of Public Health, 665 Huntington Avenue. Boston, Massachusetts 02115, USA.

**Corresponding author:** Mercedes Sotos Prieto, PhD

Department of Preventive Medicine and Public Health

School of Medicine, Universidad Autónoma de Madrid

Calle del Arzobispo Morcillo 4

28029 Madrid. SPAIN. Telephone: (+34) 914975441

E-mail: mercedes.sotos@uam.es

**Online Resource 1**. Food items constituting the 18 food groups corresponding to the Plant-based Diet Indices (modified from Satija *et al*. (2016)).

| **FOOD GROUP** | **CONTENT** | **hPDI**  **score** | **uPDI**  **score** |
| --- | --- | --- | --- |
| **Healthy plant food** | | | |
| Whole grains | Whole grain breakfast cereal, other cooked breakfast cereal, cooked oatmeal, dark bread, brown rice, other grains, bran, wheat germ, popcorn. | Positive | Reverse |
| Fruits | Raisins or grapes, prunes, bananas, cantaloupe, watermelon, fresh apples or pears, oranges, grapefruit, strawberries, blueberries, peaches or apricots or plums, fresh fruit juices. | Positive | Reverse |
| Vegetables | Tomatoes, tomato juice, tomato sauce, broccoli, cabbage, cauliflower, Brussels sprouts, carrots, mixed vegetables, yellow or winter squash, eggplant or zucchini, yams or sweet potatoes, spinach cooked, spinach raw, kale or mustard or chard greens, iceberg or head lettuce, romaine or leaf lettuce, celery, mushrooms, beets, alfalfa sprouts, garlic, corn, olives, fresh tomato juice. | Positive | Reverse |
| Nuts | Nuts, peanut or almond butter. | Positive | Reverse |
| Legumes | String beans, tofu or soybeans, beans or lentils, peas, lima beans, soy milk. | Positive | Reverse |
| Vegetable oils | Oil-based salad dressing, vegetable oil used for cooking. | Positive | Reverse |
| Coffee, tea | Coffee, decaffeinated coffee, tea, infusions. | Positive | Reverse |
| **Unhealthy plant food** | | | |
| Fruit juices | Packaged juices and juices from nectar. | Reverse | Positive |
| Refined grains | Refined grain breakfast cereal, white bread, white rice, pasta, bread, pizza mass, flour. | Reverse | Positive |
| Potatoes | French fries, baked or mashed potatoes, potato, or corn chips. | Reverse | Positive |
| Sugar-sweetened beverages | Colas with caffeine and sugar, colas without caffeine but with sugar, other carbonated beverages with sugar, noncarbonated fruit drinks with sugar, energetic drinks. | Reverse | Positive |
| Sweets and desserts | Chocolates, candy bars, candy without chocolate, cookies (home-baked and ready-made), biscuits, brownies, doughnuts, cake (home-baked and ready-made), sweet roll (home-baked and ready-made), pie (home-baked and readymade), jams or jellies or preserves or syrup or honey. | Reverse | Positive |
| **Animal food** | | | |
| Animal fat | Butter added to food, butter or lard used for cooking. | Reverse | Reverse |
| Dairy | Milk, cream, sour cream, ice cream, yogurt, cheese, cream cheese, kefir. | Reverse | Reverse |
| Egg | Eggs, omelette. | Reverse | Reverse |
| Fish & seafood | Canned tuna, dark meat fish, other fish, shrimp or lobster or scallops. | Reverse | Reverse |
| Meat | Chicken or turkey, bacon, hot dogs, processed meats, liver, hamburger, beef, pork or lamb mixed dish, beef, pork or lamb main dish, horse meat, bushmeat. | Reverse | Reverse |
| Miscellaneous animal-based foods | Pizza, chowder or cream soup, mayonnaise, or other creamy salad dressing, stuffed pasta. | Reverse | Reverse |

Abbreviations: hPDI, healthful Plant-based Diet Index; uPDI, unhealthful Plant-based Diet Index.

Food servings: whole grains 50 g, fruit 160 g, vegetable 236.6 g for green leafy and 118.3 for the rest, nuts 30 g, legumes 150 g, coffee 75 g, fruit juices 160 g, refined grains 150 g for rice and pasta and 75 for bread, potatoes 150 g, sugar-sweetened beverages 200 g, sweets 50 g, animal fat 12 g, dairy 200 g for milk and yogurt and 50 for cheese, eggs 55 g, fish and seafood 125 g, meat 125 g (42.5 if processed), miscellaneous animal-based foods servings vary depending on the composition.

**Online Resource 2**. Points assigned to quintiles of food groups for the construction of PDIs (from Satija *et al*., 2016).

|  | Quintiles | hPDI | uPDI |
| --- | --- | --- | --- |
| Healthy plant foods  (7 groups) | 1st  2nd  3rd  4th  5th | Positive scores  1  2  3  4  5 | Reverse scores  5  4  3  2  1 |
| Unhealthy plant foods  (5 groups) | 1st  2nd  3rd  4th  5th | Reverse scores  5  4  3  2  1 | Positive scores  1  2  3  4  5 |
| Animal foods  (6 groups) | 1st  2nd  3rd  4th  5th | Reverse scores  5  4  3  2  1 | Reverse scores  5  4  3  2  1 |
| Range |  | 18-90 | 18-90 |

**Online Resource 3.** Flow diagram of included participants from the Seniors ENRICA-1 cohort.

**Seniors-ENRICA-1 Cohort**

**2008-2010**

**N *=* 3,289**

**Excluded participants at baseline:**

- Missing frailty data (n = 154)
- Missing BMI (*n* = 25)
- Implausible energy intake (*n* = 7)
- Frail (*n* = 41).

**2012**

**N = 3,062**

**Excluded participants at the follow-up:**

- Dead (*n* = 108).
- Lost to follow-up (*n* = 662).
- Missing frailty data at follow-up (n = 412).

**Analysed sample**

***N* = 1,880**

**Online Resource 4.** Differences in participant’s characteristics by inclusion status into the analytical sample.

|  | **Included participants**  (N = 1880) | **Lost to follow-up**  (n = 662) | **Excluded due to**  **missing frailty data**  (n = 566) |
| --- | --- | --- | --- |
| hPDI, range (18 – 90), mean (SD) | 59.73 (5.63) | 59.49 (5.79) | 59.32 (5.59) |
| uPDI, range (18 – 90), mean (SD) | 54.85 (5.32) | 55.33 (5.80) | 55.00 (5.71) |
| Sex, women, *n* (%) | 971 (51.65) | 392 (59.21) | 312 (55.12) |
| Age, years, mean (SD) | 68.65 (6.38) | 69.57 (6.74) | 68.46 (6.19) |
| Education, *n* (%) |  |  |  |
| ≤ Primary | 997 (53.03) | 437 (66.01) | 327 (57.77) |
| Secondary | 469 (24.95) | 138 (20.85) | 132 (23.32) |
| University | 414 (22.02) | 87 (13.14) | 107 (18.90) |
| Smoking status, *n* (%) |  |  |  |
| Current | 221 (11.76) | 87 (13.14) | 58 (10.25) |
| Former | 577 (30.69) | 186 (28.10) | 140 (29.68) |
| Never | 1082 (57.55) | 389 (58.76) | 324 (60.07) |
| BMI, kg/m^2^, n (%) |  |  |  |
| < 25 | 368 (19.57) | 126 (19.57) | 111 (19.96) |
| 25 – 29.9 | 937 (49.69) | 274 (42.55) | 257 (46.22) |
| ≥ 30 | 575 (30.59) | 244 (37.89) | 188 (33.81) |
| Energy intake, kcal/day, mean (SD) | 2031 (569) | 1990 (776) | 2064 (877) |
| Alcohol intake, servings/day, mean (SD) | 1.05 (1.79) | 0.91 (1.78) | 1.08 (1.83) |
| Physical activity, METs*h/wk, mean (SD) | 21.78 (15.38) | 21.50 (14.52) | 22.46 (15.02) |
| Prevalent diseases, *n* (%) |  |  |  |
| Cardiovascular disease^a^ | 97 (5.16) | 33 (4.98) | 32 (5.65) |
| Type 2 diabetes mellitus | 284 (15.11) | 136 (20.54) | 90 (15.90) |
| Cancer | 34 (1.81) | 21 (3.17) | 9 (1.59) |
| Chronic lung disease^b^ | 143 (7.61) | 55 (8.31) | 29 (5.12) |
| Osteomuscular disease^c^ | 893 (47.50) | 352 (53.17) | 297 (52.47) |
| Depression | 138 (7.34) | 76 (11.48) | 53 (9.36) |
| Medicines per day, n (%) |  |  |  |
| 0 | 504 (26.81) | 152 (22.96) | 165 (29.15) |
| 1 – 3 | 993 (52.82) | 359 (54.23) | 274 (48.41) |
| 4 – 6 | 332 (17.66) | 128 (19.34) | 109 (19.26) |
| > 6 | 51 (2.71) | 23 (3.47) | 18 (3.18) |

Abbreviations: p, points; SD, standard deviation; BMI, body mass index.

a Including myocardial infarction, stroke, and heart failure.

b Including asthma and chronic obstructive pulmonary disease.

c Including osteoarthritis, rheumatoid arthritis, and hip fracture.

**Online Resource 5**. Secondary analyses. Odds ratios (95% CI) for the occurrence of frailty by tertiles of plant-based diet indices over 3.3 years of follow-up in the Seniors ENRICA-1 cohort.

|  | Tertile 1 (lowest) | Tertile 2 | Tertile 3 (highest) | *P*-trend |
| --- | --- | --- | --- | --- |
| **Excluding pre-frail participants (N = 972)** | | | | |
| n/N  hPDI | 41/234  Ref. | 55/388  0.56 (0.30 – 1.07) | 40/350  0.40 (0.20 – 0.82) | **0.014** |
| n/N  uPDI | 37/464  Ref. | 51/321  1.67 (0.90 – 3.07) | 48/187  3.84 (1.96 – 7.50) | **< 0.001** |
| **Excluding participants with prevalent diseases (N = 1294)** | | | | |
| n/N  hPDI | 18/293  Ref. | 31/528  0.65 (0.32 – 1.32) | 20/473  0.40 (0.18 – 0.88) | **0.021** |
| n/N  uPDI | 17/610  Ref. | 24/442  1.60 (0.80 – 3.21) | 28/242  3.03 (1.50 – 6.14) | **0.002** |
| **Among participants self-reportedly healthy and independent in instrumental activities of daily living (n = 1204)** | | | | |
| n/N  hPDI | 14/281  Ref. | 12/479  0.46 (0.18 – 1.14) | 8/444  0.29 (0.10 – 0.87) | **0.025** |
| n/N  uPDI | 9/611  Ref. | 14/386  **2.78 (1.07 – 7.20)** | 11/207  3.54 (1.28 – 9.78) | **0.011** |
| **Excluding weight loss from the definition of frailty (N = 1880)** | | | | |
| n/N  hPDI | 41/429  Ref. | 54/765  0.62 (0.35 – 1.10) | 40/686  0.53 (0.28 – 0.99) | 0.058 |
| n/N  uPDI | 37/879  Ref. | 51/639  1.41 (0.82 – 2.44) | 48/362  2.09 (1.17 – 3.74) | **0.013** |
| **Alternative hPDI: considering fish, eggs and dairy as healthy products (N = 1880)** | | | | |
| Range | 41 – 55 p | 56 – 61 p | 62 – 80 p |  |
| n/N | 30/309 | 54/701 | 52/870 |  |
| Modified hPDI | Ref. | 0.65 (0.38 – 1.12) | 0.40 (0.23 – 0.68) | **< 0.001** |

Abbreviations: CI, confidence interval; Ref., reference.

All models: adjusted for sex, age, educational level (primary, secondary, university), alcohol consumption, smoking status (current, former, never), BMI (< 25, 25 – 29.9, ≥ 30), energy intake, physical activity, prevalent diseases (type 2 diabetes mellitus, cardiovascular disease [myocardial infarction, stroke, or heart failure], chronic lung disease [asthma or chronic obstructive pulmonary disease], osteomuscular disease [osteoarthritis, rheumatoid arthritis or hip fracture], cancer and depression), number of medicines consumed (0, 1-3, 4-6, > 6).
